# Long Noncoding RNA HCP5 Contributes to Nasopharyngeal Carcinoma Progression by Targeting MicroRNA-128-3p

**DOI:** 10.1155/2022/5740857

**Published:** 2022-05-12

**Authors:** Gangyong Miao, Bin Liu, Keji Ling, Tao Peng, En Zhou, Sijun Xie, Zhiqiang Tan

**Affiliations:** ^1^Department of Otorhinolaryngology Head and Neck Surgery, Hunan Provincial People's Hospital, Changsha 410005, China; ^2^Department of Ophthalmology, 2nd Affiliated Hospital of Xiangya College, Central South University, Changsha, China

## Abstract

**Aim:**

To determine the role and underlying mechanism of lncRNA HCP5 in nasopharyngeal carcinoma (NPC).

**Method:**

The expression of HCP5 and miR-128-3p was assessed by qRT-PCR. CCK-8, EdU staining, and transwell were performed to determine cell progression. A nude mouse xenograft tumor model was carried out to detect the role of HCP5 *in vivo*. The luciferase assay was performed to confirm the function between lncRNA HCP5 and miR-128-3p.

**Results:**

The increased level of HCP5 was observed in NPC tissues. Silencing of HCP5 prevented tumor progression *in vitro* and *in vivo*. The luciferase assay verified that HCP5 could bind with miR-128-3p. Furthermore, forced expression of miR-128-3p could prevent the function of HCP5 on NPC cells.

**Conclusion:**

lncRNA HCP5 could regulate NPC cell progression *via* sponging miR-128-3p, which might serve as a potential therapy target of NPC.

## 1. Introduction

Nasopharyngeal carcinoma (NPC) is the most common craniofacial malignant tumor with unique geographical, etiological, and biological characteristics. At present, the main treatment of NPC is radiotherapy combined with comprehensive clinical treatment, and the late discovery and prevalent metastasis are the main reasons for the poor prognosis and the decline of the survival rate of NPC [1, 2]. More and more data show that the noncoding RNA in tumor tissues can be used as a new molecular marker for early diagnosis of tumors and to judge the treatment and prognosis of the disease according to its expression level [3, 4]. Noncoding RNAs can also inhibit or promote tumor growth through a series of mechanisms, such as transcriptional regulation and epigenetic regulation [5].

lncRNAs are a kind of noncoding RNA, which is more than 200 bp and regulates genes. lncRNAs play an essential role in regulating epigenetic, transcriptional, and posttranscriptional levels [6, 7]. lncRNAs regulate some biological processes of cells through interaction with DNA, RNA, and protein molecules, including epigenetic regulation, X chromosome silencing, genomic imprinting and staining, cytoplasmic modification, and nuclear-cytoplasmic transport, mRNA splicing, transcription, and translation regulation [8]. The high expression of lncRNA Hotair could promote the metastasis of different cancers. In NPC, Hotair could promote tumor development by directly activating the transcription of angiogenic factor VEGFA and upregulating the expression of VEGFA and Ang2 mediated by GRP78 or through FASN [9]. Zhuang found that low expression of lncRNA HNF1A-AS could weaken NPC cell progression and proved that HNF1A-AS is related to the epithelial-mesenchymal transformation (EMT) pathway [10]. Although lncRNA and miRNA may play an independent role in the progression of NPC, these two kinds of ncRNA could also affect the development of NPC. It has been found that lncRNA H19 regulated the EZH2 level by preventing miR-630 and then simulated the progression of nasopharyngeal carcinoma [11].

In this research, we detected the expression level of lncRNA HCP5 in NPC tissues. Moreover, we analyzed the molecules of lncRNA HCP5 affecting the progression of NPC cells to indicate a novel NPC therapy target.

## 2. Results

### 2.1. Silencing of HCP5 Inhibits Tumor Progression in NPC Cells

**Figure 1:**
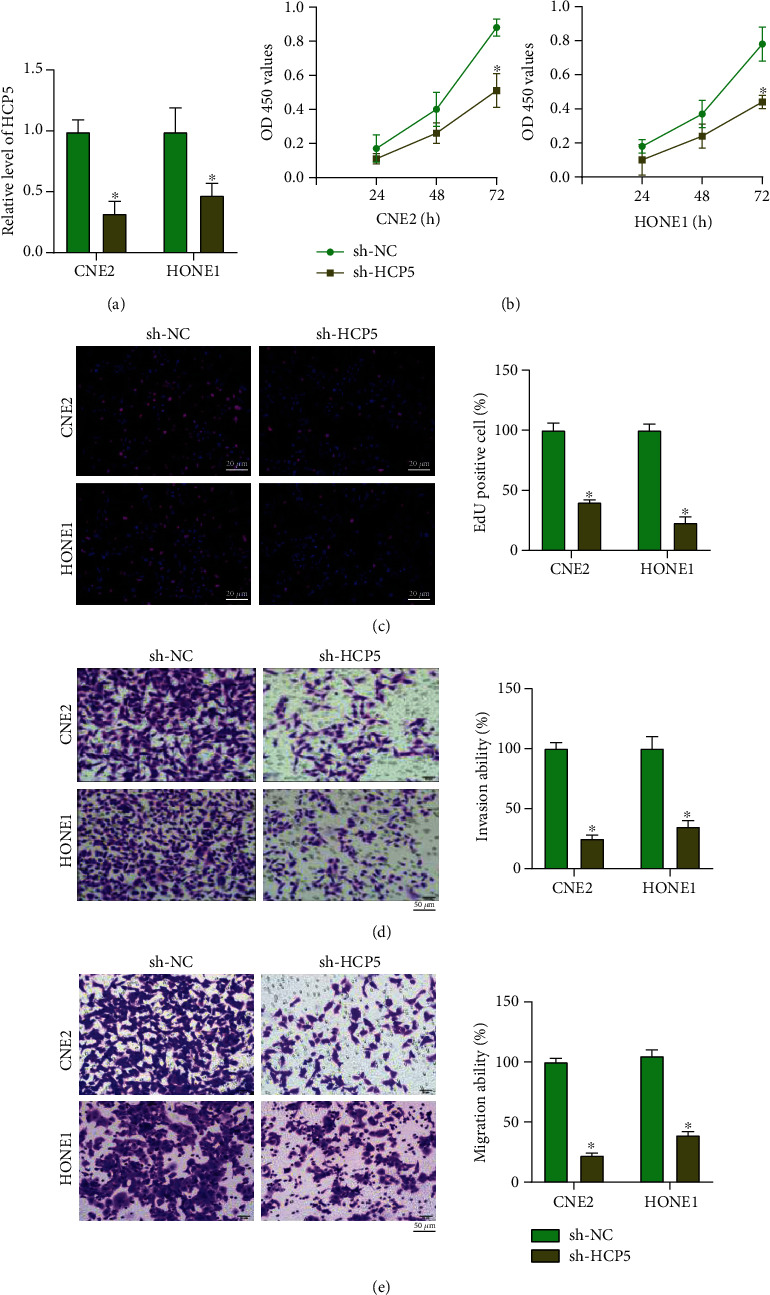
Knockdown of HCP5 inhibits tumor progression in NPC cells. (a) The expression of HCP5 in NPC cells. *n* = 6,  ^∗^*P* < 0.05. (b) CCK8 assay was used to detect cell viability in each group. *n* = 6, ^∗^*P* < 0.05. (c) EdU assay was performed to detect cell proliferation ability in each group. *n* = 6,  ^∗^*P* < 0.05. (d, e) Cell invasion and migration ability were analyzed by transwell assay. Scale bar, 50 *μ*m. *n* = 6,  ^∗^*P* < 0.05.

We constructed shRNA for inhibiting the expression level of HCP5 in different NPC cell lines. The knockdown efficiency was confirmed in NPC cells (Figure 1(a)). The CCK-8 assay was performed to detect the cell viability in NPC cells. We observed that sh-HCP5 inhibited cell viability in CNE2 and HONE1 cells (Figure 1(b)). The EdU staining assay showed that sh-HCP5 blocked cell proliferation in NPC cells (Figure 1(c)). Meanwhile, sh-HCP5 prevented invasion and migration ability in CNE2 and HONE1 cells (Figures 1(d) and 1(e)). Taken together, silencing of HCP5 controlled tumor progression by inhibiting tumor progression in NPC cells.

### 2.2. HCP5 Could Interact with miR-128-3p

**Figure 2:**
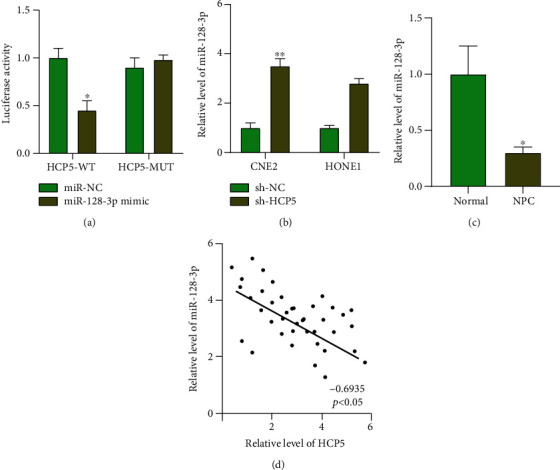
HCP5 could interact with miR-128-3p. (a) Targeting prediction results of miR-128-3p and HCP5. The results of luciferase assay. *n* = 3,  ^∗^*P* < 0.05. (b) The level of miR-128-3p in NPC cells after sh-HCP5 transfection. *n* = 6,  ^∗^*P* < 0.05,  ^∗∗^*P* < 0.01. (c) The level of miR-128-3p in NPC tissues. *n* = 40,  ^∗^*P* < 0.05. (d) The relationship between miR-128-3p and HCP5 NPC tissues. *n* = 40,  ^∗^*P* < 0.05.

Bioinformatics website predicted that miR-128-3p could interact with HCP5, and the predicted binding sites are shown in Figure 2(a) (upper). The luciferase assay demonstrated that miR-128-3p mimic cotransfected with HCP5 wild type (HCP5-WT) decreased luciferase activity, while not HCP5 mutant (HCP5-MUT); the results determined that miR-128-3p could interact with HCP5 (Figure 2(a)). Silencing of HCP5 could inhibit the level of miR-128-3p in NPC cells (Figure 2(b)). We found that the level of miR-128-3p was decreased in NPC tissues (Figure 2(c)). Further, qPCR analyzes a negative relationship between HCP5 and miR-128-3p (Figure 2(d)).

### 2.3. miR-128-3p Prevents the Function of HCP5 on Tumor Progression

**Figure 3:**
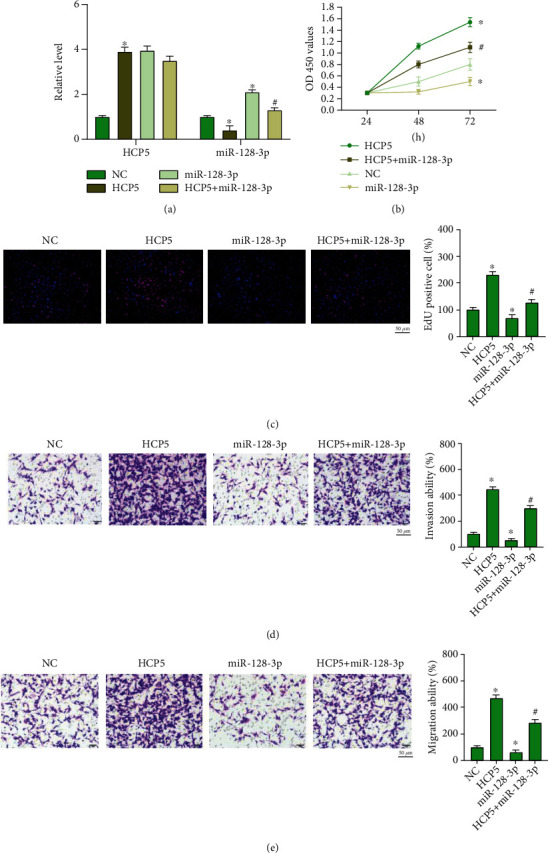
miR-128-3p prevents HCP5 function on NPC progression. (a) The expression of HCP5 and miR-128-3p in CNE2 cells. *n* = 4,  ^∗^*P* < 0.05*vs.* NC, ^#^*P* < 0.05*vs.* HCP5. (b) The cell viability was analyzed by CCK-8 assay. *n* = 4,  ^∗^*P* < 0.05*vs.* NC, ^#^*P* < 0.05*vs.* HCP5. (c) EdU assay was performed to detect cell proliferation ability in each group. *n* = 4,  ^∗^*P* < 0.05*vs.* NC, ^#^*P* < 0.05*vs.* HCP5. (d, e) Cell invasion and migration ability were analyzed by transwell assay. Scale bar, 50 *μ*m. *n* = 4,  ^∗^*P* < 0.05*vs.* NC, ^#^*P* < 0.05*vs.* HCP5.

**Figure 4:**
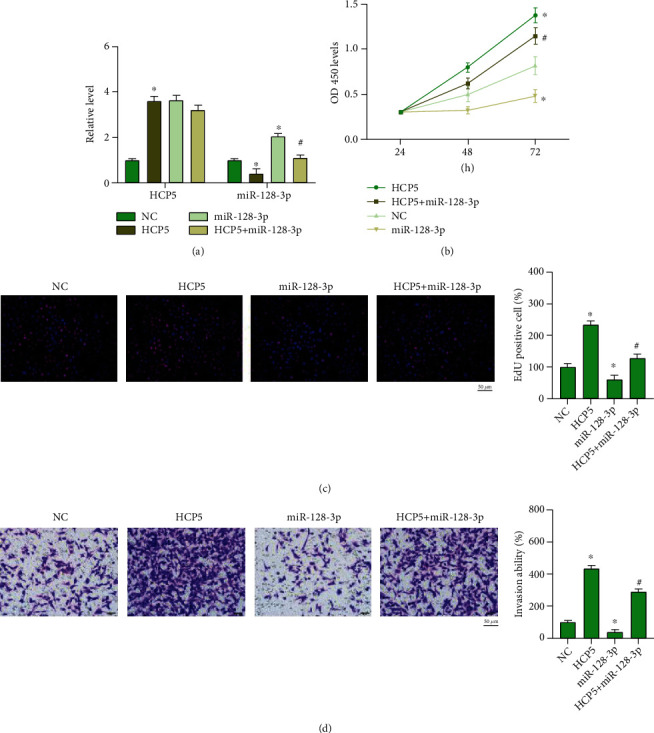
miR-128-3p prevents HCP5 function on NPC progression. (a) The expression of HCP5 and miR-128-3p HONE1 cells. *n* = 4,  ^∗^*P* < 0.05*vs.* NC, ^#^*P* < 0.05*vs.* HCP5. (b) The cell viability was analyzed by CCK-8 assay. *n* = 4,  ^∗^*P* < 0.05*vs.* NC, ^#^*P* < 0.05*vs.* HCP5. (c) EdU assay was performed to detect cell proliferation ability in each group. *n* = 4,  ^∗^*P* < 0.05*vs.* NC, ^#^*P* < 0.05*vs.* HCP5. Scale bar, 50 *μ*m. (d) Cell invasion ability was analyzed by transwell assay. Scale bar, 50 *μ*m. *n* = 4, ^∗^*P* < 0.05*vs.* NC, ^#^*P* < 0.05*vs.* HCP5.

**Figure 5:**
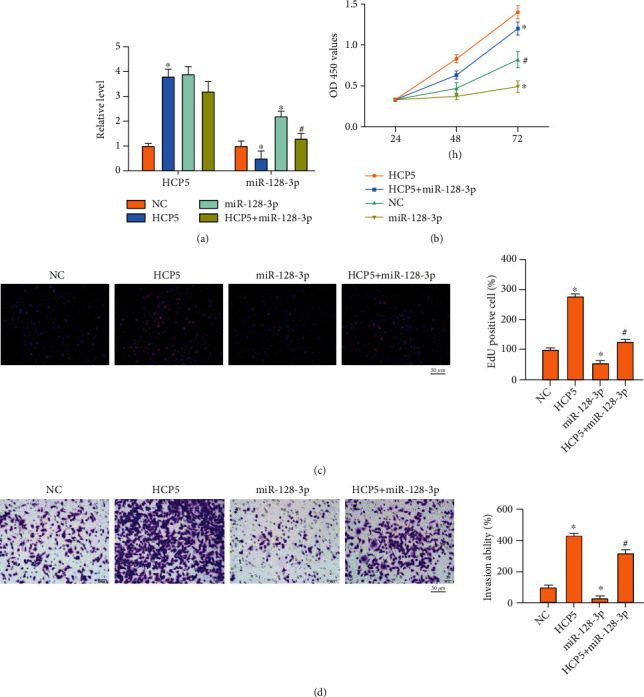
miR-128-3p prevents HCP5 function on NPC progression. (a) The expression of HCP5 and miR-128-3p SUNE-1 cells. ^∗^*P* < 0.05*vs.* NC, ^#^*P* < 0.05*vs.* HCP5. (b) The cell viability was analyzed by CCK-8 assay. *n* = 4, ^∗^*P* < 0.05*vs.* NC, ^#^*P* < 0.05*vs.* HCP5. (c) EdU assay was performed to detect cell proliferation ability in each group. *n* = 4, ^∗^*P* < 0.05*vs.* NC, ^#^*P* < 0.05*vs.* HCP5. Scale bar, 50 *μ*m. (d) Cell invasion ability was analyzed by transwell assay. Scale bar, 50 *μ*m. *n* = 4, ^∗^*P* < 0.05*vs.* NC, ^#^*P* < 0.05*vs.* HCP5.

Further, we constructed HCP5 plasmid and miR-128-3p mimic, cotransfected into CNE2, HONE1, and SUNE-1 cells (Figures 3(a), 4(a), and 5(a)). HCP5 induced and miR-128-3p repressed cell proliferation, in which the effect of HCP5 was abolished by miR-128-3p overexpression (Figures 3(b), 4(b), and 5(b)). Meanwhile, Edu staining showed that HCP5 facilitated and miR-128-3p inhibited cell proliferation, and miR-128-3p prevented the function of HCP5 on CNE2, HONE1, and SUNE-1 cells (Figures 3(c), 4(c), and 5(c)). HCP5 also triggered migration and invasion, while miR-128-3p blocked HCP5 function in CNE2, HONE1, and SUNE-1 cells (Figures 3(d), 3(e), 4(d), and 5(d)). Taken together, miR-128-3p would be a target of HCP5 and is involved in HCP regulating NPC progression.

### 2.4. HCP5 Blockade Inhibits Tumor Growth *In Vivo*

**Figure 6:**
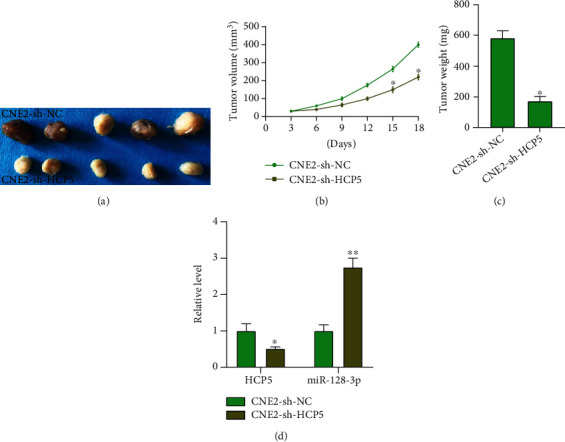
Knockdown of HCP5 could inhibit tumor growth in vivo. (a) The tumor image in each group. *n* = 5. (b, c) The tumor volume and weight were performed in each group. *n* = 5, ^∗^*P* < 0.05. (d) The expression of HCP5 and miR-128-3p in tumor tissues. *n* = 5,  ^∗^*P* < 0.05,  ^∗∗^*P* < 0.01.

We constructed stable HCP5 low expression CNE2 cells (CNE2-sh-HCP5) and normal CNE2 cells (CNE2-sh-NC) and injected them into nude mice to observe the tumor growth. The tumor image is shown in Figure 6(a). Silencing of HCP5 prevented tumor volume and weight (Figures 6(b) and 6(c)). The decreased expression of HCP5 and increased expression of miR-128-3p were observed in tumor tissues (Figure 6(d)).

## 3. Discussion

Finding new molecular targets for the growth and metastasis of NPC is the direction of clinical treatment of NPC in the future [12]. More and more studies have shown that lncRNA can play the role of epigenetic regulation, transcriptional regulation, and posttranscriptional regulation and participate in the occurrence and development of diseases [13–15]. The function of lncRNA on the proliferation and metastasis of NPC cells has been widely concerned.

lncRNA HCP5 is overexpressed in follicular thyroid carcinoma and could induce tumor development [16]. lncRNA HCP5 adjustable MACC1 triggers cervical cancer progression. Studies have shown that the level of HCP5 is increased in glioma tissues and U87 and U251 cells. Knockdown of HCP5 can reduce cell progression and induce apoptosis [17]. It has been reported that lncRNA HCP5 is highly expressed in triple-negative breast cancer. Knocking down HCP5 can regulate miR-219a-5p to inhibit triple-negative breast cancer cell growth and induce apoptosis [18]. lncRNA HCP5 induces the development of lung adenocarcinoma cells by targeting miR-203 [19]. Silencing HCP5 prevents osteosarcoma development [20, 21].

In this experiment, we used qRT-PCR technology to analyze the level of HCP5 in NPC tissues. The data performed that HCP5 was elevated in NPC tissues. Through the cell function experiment, we concluded that the high expression of HCP5 could enhance NPC cell progression; silencing of HPC5 inhibits NPC development *in vitro* and *in vivo*. Our experiments proved that HCP5 could promote the process of NPC by regulating the proliferation, migration, and invasion of NPC cells.

Recent research revealed that lncRNA could specifically bind to miRNA as a competitive endogenous RNA and downregulate the activity of miRNA, thus indirectly upregulating the expression of miRNA target genes [22, 23]. Bioinformatics methods showed that HCP5 might target the regulation of miR-128-3p. Forced expression of HCP5 in NPC cells promoted cell progression, while these functions could be blocked by overexpression of miR-128-3p. These data indicated that HCP5 could regulate NPC progression via targeting miR-128-3p. Interestingly, we found that miR-128-3p upregulated the expression of HCP5 in the cells. The potential mechanism of the feedback regulation effect of miR-128-3p on HCP5 is needed to be explored in future investigations. Moreover, HCP5 is able to target multiple factors in several cancers and other diseases. For example, it has been reported that HCP5 regulates premature ovarian insufficiency through transcriptionally modulating MSH5 to mediate DNA damage repair by YB1 [24]. HCP5 inhibits the development of skin cutaneous melanoma through modulating miR-12/RARRES3 [25]. HCP5 promotes cisplatin resistance by targeting PTEN in triple-negative breast cancer [18]. HCP5 enhances epithelial-mesenchymal transition by activating ZEB1 and targeting miR-139-5p in colorectal cancer [26]. SMAD3-responsive HCP5 contributes to lung adenocarcinoma metastasis by targeting miR-203/SNAI signaling [19]. HCP5 enhances cell proliferation of prostate cancer by targeting the miR-4656/CEMIP axis [27]. The potential function and correlation of HCP5 with these targets in the regulation of NPC should be explored in future investigations.

## 4. Conclusion

Here, the upregulation of HCP5 was observed in NPC tissues. We demonstrated that HPC5 could induce NPC cell proliferation, migration, and invasion. Further, HCP5 could interact with miR-128-3p in NPC cells and regulate cell progression, which could be a novel therapy target of NPC.

## 5. Materials and Methods

### 5.1. Cell Culture

NPC cell lines (CNE-2Z, HONE-1, and SUNE-1) were purchased from a typical culture preservation center in China. The cells were cultured in RPMI1640 medium (containing 10% fetal bovine serum) and cultured at 37°C and 5% CO_2_ incubator. The cells in the logarithmic growth phase were transfected, and the negative control plasmid with the meaningless sequence was used as the negative control group, and the transfection operation was carried out according to the Lipofectamine™ 2000 instructions.

### 5.2. qRT-PCR

The total RNA of tissue or cell was extracted by the TRIzol method and reverse transcribed into cDNA; then, the operation was carried out according to the instructions of the qPCR kit. The following thermocycling conditions were used for qPCR: initial denaturation at 95°C for 30 sec; followed by 39 cycles at 95°C for 5 sec and 60°C for 30 sec; and a final extension at 72°C for 5 min. The 2^-*ΔΔ*Cq^ method was used. The primer sequences are as follows: HCP5 forward: 5′-TCTCCTTCTGCCCATCACTTG-3′, reverse: 5′-AACCCTCCTCCTGCTGTTCTC-3′; miR-128-3p forward: 5′-GGTCACAGTGAACCGGTC-3′, reverse: 5′-GTGCAGGGTCCGAGGT-3′; U6 forward: 5′-CTCGCTTCGGCAGCACATATACT-3′, reverse: 5′-ACGCTTCACGAATTTGCGTGTC-3′; and GAPDH forward: 5′-AACGGATTTGGTCGTATTGGG-3′, reverse: 5′-CCTGGAAGATGGTGATGGGAT-3′.

### 5.3. EdU Staining

The proliferation of cells was evaluated by the EdU kit (Beyotime, China, C0078S). The logarithmic phase cells were inoculated into a 24-well plate according to the density of 5 × 10^4^ cells per well. After the cells were synchronized with serum-free medium, the 10 *μ*mol·L-1 EdU reagent was added to the cells according to the instructions of the EdU fluorescence staining cell proliferation kit. The fixed solution was washed out with PBS, then incubated with Apollo dye solution for 30 min, PBS at room temperature, and stained with DAPI for 5 min. The fluorescence images of 5 visual fields were randomly obtained by the IX73 fluorescence microscope, and the EdU-positive cells were counted by ImageJ software (NIH, v1.8.0).

### 5.4. Transwell Assay

Transwell assays analyzed the invasion and migration by using a transwell plate (Corning, USA) according to the manufacturer's instruction. To analyze the cell migration, the cells were cultured for 24 hours and resuspended by serum-free culture medium, then plated into the apical chamber of transwell at a density of 5 × 10^3^ cells/well. The culture medium was made up to 150 *μ*L, and the basolateral chamber was added with 600 *μ*L complete culture medium. After 24 hours, culture at 37°C and 5% CO_2_, the cells were fixed through 4% paraformaldehyde for 10 minutes, stained with crystal violet dye for 20 minutes, followed by the analysis using the intelligent biological navigator (Olympus, Tokyo, Japan). The migrated cells were recorded and calculated by using the ImageJ software.

To analyze the cell invasion, Matrigel was melted overnight at 4°C and diluted by presold serum-free culture medium (dilution ratio 1 : 8). The medium (50 *μ*L) was plated into the transwell polycarbonate membrane with a pore diameter of 8 *μ*m, making all the wells covered by Matrigel at 37°C for 2 hours. The cells were cultured for 24 hours and resuspended by serum-free culture medium, then plated into the apical chamber of transwell at 1 × 10^5^ cells/well; the medium was made up to 150 *μ*L. The basolateral chamber was added with 600 *μ*L complete medium with 50% FBS. After 24 hours, the cells were fixed using 4% paraformaldehyde for 15 minutes, stained with crystal violet dye for 10 minutes. The invaded cells were analyzed and calculated by using the ImageJ software.

### 5.5. Luciferase Reporter

According to Starbase 3.0 software, HCP5 has a highly conserved binding site of miR-128-3p. Luciferase reporter plasmid, HCP5 wild type (WT, UCCCAGGCCCUCCACUGUGA), and HCP5 mutant (MUT, UCCCAGGCCCUCGCGAAGCA) plasmid were synthesized and identified by Guangzhou Ruibo Biotechnology Co., Ltd. In the luciferase reporter gene assay, cells were cotransfected with HCP5-WT or HCP5-MUT, miR-128-3p mimic, or NC with Lipofectamine 2000. 48 hours after transfection, the cells were harvested and lysed. The luciferase activity was analyzed by the Dual-Glo® luciferase assay system and normalized by firefly luciferase activity.

### 5.6. Animal Experiment

The experiment on subcutaneous tumorigenesis was carried out in 6-week-old male nude mice. The cells growing in the logarithmic phase were collected and counted by trypsin digestion. The cells suspended in normal saline were injected into the groin or armpit of nude mice. The growth curve of subcutaneous tumors in nude mice was recorded every 3 days. After 18 days of feeding, the animals were killed, and tumors were removed and weighed. After the tumor was frozen in liquid nitrogen, it was stored in a -80°C refrigerator for further experiment. The animal study was reviewed and approved by our hospital.

### 5.7. Statistical Methods

The statistical analysis was carried out by Prism 8.0. One-way ANOVA was used for the comparison between multiple groups, and *t*-test was used for the comparison between two groups. All the data were expressed in terms of the mean ± SEM, and the difference (*P* < 0.05) was statistically significant.

## Data Availability

The datasets used during the present study are available from the corresponding author upon reasonable request.

## References

[1] Zhang Y., Chen L., Hu G. (2019). Gemcitabine and cisplatin induction chemotherapy in nasopharyngeal carcinoma. *The New England journal of medicine*.

[2] Sun Y., Li W., Chen N. (2016). Induction chemotherapy plus concurrent chemoradiotherapy versus concurrent chemoradiotherapy alone in locoregionally advanced nasopharyngeal carcinoma: a phase 3, multicentre, randomised controlled trial. *The Lancet Oncology*.

[3] Anastasiadou E., Jacob L., Slack F. (2018). Non-coding RNA networks in cancer. *Nature reviews Cancer*.

[4] Huang H., Weng H., Chen J. (2020). m^6^A modification in coding and non-coding RNAs: roles and therapeutic implications in cancer. *Cancer Cell*.

[5] Goodall G., Wickramasinghe V. (2021). RNA in cancer. *Cancer*.

[6] Schmitt A., Chang H. (2016). Long noncoding RNAs in cancer pathways. *Cancer Cell*.

[7] Hu Q., Ye Y., Chan L. (2019). Oncogenic lncRNA downregulates cancer cell antigen presentation and intrinsic tumor suppression. *Nature Immunology*.

[8] Jiang S., Cheng S., Ren L. (2019). An expanded landscape of human long noncoding RNA. *Nucleic acids research*.

[9] Ma D., Yuan L., Lin L. (2017). LncRNA HOTAIR contributes to the tumorigenesis of nasopharyngeal carcinoma via up-regulating FASN. *European review for medical and pharmacological sciences*.

[10] Zhuang K., Wu Q., Jin C., Yuan H., Cheng J. (2016). Long non-coding RNA HNF1A-AS is upregulated and promotes cell proliferation and metastasis in nasopharyngeal carcinoma. *Cancer biomarkers: section A of Disease markers*.

[11] Li X., Lin Y., Yang X., Wu X., He X. (2016). Long noncoding RNA H19 regulates EZH2 expression by interacting with miR-630 and promotes cell invasion in nasopharyngeal carcinoma. *Biochemical and biophysical research communications*.

[12] Hui E., Li W., Ma B. (2020). Integrating postradiotherapy plasma Epstein-Barr virus DNA and TNM stage for risk stratification of nasopharyngeal carcinoma to adjuvant therapy. *Annals of oncology: official journal of the European Society for Medical Oncology*.

[13] Wang Y., Chen W., Lian J. (2020). The lncRNA PVT1 regulates nasopharyngeal carcinoma cell proliferation via activating the KAT2A acetyltransferase and stabilizing HIF-1*α*. *Cell death and differentiation*.

[14] Guo Z., Wang Y., Xu H. (2021). LncRNA linc00312 suppresses radiotherapy resistance by targeting DNA-PKcs and impairing DNA damage repair in nasopharyngeal carcinoma. *Cell death & disease*.

[15] Wang Z., Cheng Y., Zhu Y. (2020). Long non-coding RNA FOXD1-AS1 promotes the progression and glycolysis of nasopharyngeal carcinoma by sustaining FOXD1 expression. *American journal of cancer research*.

[16] Liang L., Xu J., Wang M. (2018). LncRNA HCP5 promotes follicular thyroid carcinoma progression via miRNAs sponge. *Cell death & disease*.

[17] Yu Y., Shen H., Fang D., Meng Q., Xin Y. (2018). LncRNA HCP5 promotes the development of cervical cancer by regulating MACC1 via suppression of microRNA-15a. *European review for medical and pharmacological sciences*.

[18] Wu J., Chen H., Ye M. (2019). Downregulation of long noncoding RNA HCP5 contributes to cisplatin resistance in human triple-negative breast cancer via regulation of PTEN expression. *Biomedicine & pharmacotherapy = Biomedecine & pharmacotherapie*.

[19] Jiang L., Wang R., Fang L. (2019). HCP5 is a SMAD3-responsive long non-coding RNA that promotes lung adenocarcinoma metastasis via miR-203/SNAI axis. *Theranostics*.

[20] Tu Y., Cai Q., Zhu X., Xu M. (2021). Down-regulation of HCP5 inhibits cell proliferation, migration, and invasion through regulating EPHA7 by competitively binding miR-101 in osteosarcoma. *Revista brasileira de pesquisas medicas e biologicas*.

[21] Zhao W., Li L. (2019). SP1-induced upregulation of long non-coding RNA HCP5 promotes the development of osteosarcoma. *Pathology, research and practice*.

[22] Sebastian-delaCruz M., Gonzalez-Moro I., Olazagoitia-Garmendia A., Castellanos-Rubio A., Santin I. (2021). The role of lncRNAs in gene expression regulation through mRNA stabilization. *RNA*.

[23] Beermann J., Piccoli M., Viereck J., Thum T. (2016). Non-coding RNAs in development and disease: background, mechanisms, and therapeutic approaches. *Physiological Reviews*.

[24] Wang X., Zhang X., Dang Y. (2020). Long noncoding RNA HCP5 participates in premature ovarian insufficiency by transcriptionally regulating MSH5 and DNA damage repair via YB1. *Nucleic Acids Research*.

[25] Wei X., Gu X., Ma M., Lou C. (2019). Long noncoding RNA HCP5 suppresses skin cutaneous melanoma development by regulating *RARRES3* gene expression via sponging miR-12. *Oncotargets and Therapy*.

[26] Yang C., Sun J., Liu W. (2019). Long noncoding RNA HCP5 contributes to epithelial-mesenchymal transition in colorectal cancer through ZEB1 activation and interacting with miR-139-5p. *American Journal of Translational Research*.

[27] Hu R., Lu Z. (2020). Long non‑coding RNA HCP5 promotes prostate cancer cell proliferation by acting as the sponge of miR‑4656 to modulate CEMIP expression. *Oncology Reports*.

